# Deodorizing India-Rubber

**Published:** 1867-07

**Authors:** 


### SELECTED ARTICLES.

### ARTICLE V.

### *Deodorizing India-Rubber.*

The extremely disagreeable odor attaching to india-rubber manufactures, and the power possessed by them of imparting a nauseous taste to liquids or other substances, has long been a difficulty in the way of its use for many purposes for which india-rubber is peculiarly adapted. To obviate this evil many expedients have been resorted to, but none hitherto with perfect success, and this on account of the strong tendency which india-rubber has to acquire and retain odors. The new process, invented by Mr. S. Bourne, depends upon the still greater affinity possessed by charcoal, especially animal charcoal, for all kinds of odors, and its great capacity for the absorption of gases. The practical difficulty lies in so using the charcoal as not to injuriously affect the articles with which it may be brought into contact, and this has now been overcome by very simple means.

The mode of application necessarily varies according to the description of articles which are thus treated. Generally speaking, they are laid in shelves or trays in a hot chamber, with a thin stratum of charcoal beneath and on top, and exposed to a temperature of from 120 to 180 degrees for from three to six hours, after which they are removed from the charcoal, having sustained no other alteration than the all important one of being rendered devoid of smell and incapable of imparting any taste to liquids or other substances they may touch. Under proper management the most delicate textures can be thus dealt with without being impaired either in substance or appearance. The most convenient mode of applying heat is by hot water or by steam surrounding the vessel or chamber in which they are placed. One very considerable advantage of this process is, that for a large number of vulcanized articles it can be carried on in co-operation with the heating or curing by which the vulcanization is effected, and they leave the chamber at once free from odor. It is equally applicable to india-rubber in sheet, spread fabrics, or the garments or other articles made therefrom when fully made up, such as the ordinary "macintosh" clothing, air and water cushions, etc. The use of this process enables the inventor to produce his "flexible diaphragms" (which were first brought before the public at the Dublin Exhibition, where they obtained a prize medal) in so pure a state that they may at once be used with the most delicate wines and other liquids. The diaphragm itself is a contrivance for the division of casks or other vessels into two separate chambers, by means of a flexible partition, which fits to the upper or lower part of the vessel alternately, or into any intermediate position, so that whatever the quantity of liquor contained within it, the air (though still exercising its pressure through the medium of the diaphragm) is separated from it by an impervious shield, and thus the injurious effects of exposure to atmospheric influence are altogether avoided, and any portion of the liquor may be withdrawn at pleasure, and as often as may be, without any admission of air to the remaining portion. In this way vessels of wine and beer are stated to have been actually kept in constant use for six and twelve months without any fermentation or formation of acid resulting. It is equally applicable to other liquids for domestic use or for medical or scientific purposes, the fluid remaining as completely secured as if the vessel were actually full.

An adjunct to this invention, and which admits also of independent use, is in the elastic valves, in two varieties—the one for giving vent to the products of fermentation, when desired; such as the carbonic acid gas generated by malt liquors, etc., the other for giving admission to air so as to enable the liquid to flow through the tap or other orifice. In the one case a circular disk of vulcanized indiarubber is made to cover a small opening through which the gas is free to escape, but meets in its passage with the india-rubber, which being forcibly held down round its edge is at liberty to become distended, and in so distending opens a number of very minute holes, which have been pierced through its surface. When the pressure is removed, the disk again becomes flat, and its orifices shut. The degree of pressure to be sustained before these perforations open is perfectly under control, and may be adjusted to any required degree.

In the other form a small cylinder of india-rubber, closed at its lower end, is drawn over a corresponding cylinder of wood with a hole through its centre, and then tightly bound at its upper edge. The india-rubber has a number of slits made in its substance, which (when any orifice through which the liquor may flow is opened) receives the pressure of air, and yielding to this, opens, so as to let the air enter the vessel in exactly the same extent as the liquor is withdrawn. When the flow of liquor is so stopped, the edges of the slits become drawn together, so as to prevent any escape of liquor or gas in a wrong direction. Should there be any pressure from within upon the surface of the india-rubber, this will only tend to a more perfect closing of the slits, and, thus, while affording sufficient ingress, altogether restrain egress.—*London Pharm. Journ., February*, 1867, from *Journ. Soc. Arts.*

---

